# Felonious Physicians

**Published:** 1898-07-09

**Authors:** 


### FELONIOUS PHYSICIANS.

It appears that in New York State there is a law which forbids a physician who has been convicted of a felony to practice his profession after being liberated from prison. Some, however, have held that it was not only unjust, but unconstitutional, that a new punishment should thus be added to the sentence pronounced by the courts and served by the convict; nor can it be doubted that there is much to be said for those who object to a special and extra punishment being inflicted on physicians which does not fall on other classes committing precisely the same crime. No doubt with us custom does much the same, so far as better class practice is concerned, as is done in New York State by law, but we do not absolutely take away a man's means of earning his living —a man "goes under," but he need not absolutely starve. The Supreme Court, however, has recently upheld the constitutionality of this New York State law, and, that being so, we can only hope that its existence will be borne in mind by judges when they consider their sentences, for it is obviously unfair that a man whose punishment is made by law to extend through the whole term of his life should be given the same length of imprisonment as one who, as in the case of many a labourer, steps out of gaol a free man, as able as ever to earn his living, and, too often it is to be feared, not materially reduced in the social estimation of his fellows.

